# Variation in Array Size, Monomer Composition and Expression of the Macrosatellite DXZ4

**DOI:** 10.1371/journal.pone.0018969

**Published:** 2011-04-22

**Authors:** Deanna C. Tremblay, Shawn Moseley, Brian P. Chadwick

**Affiliations:** Department of Biological Science, Florida State University, Tallahassee, Florida, United States of America; Oregon State University, United States of America

## Abstract

Macrosatellites are some of the most polymorphic regions of the human genome, yet many remain uncharacterized despite the association of some arrays with disease susceptibility. This study sought to explore the polymorphic nature of the X-linked macrosatellite DXZ4. Four aspects of DXZ4 were explored in detail, including tandem repeat copy number variation, array instability, monomer sequence polymorphism and array expression. DXZ4 arrays contained between 12 and 100 3.0 kb repeat units with an average array containing 57. Monomers were confirmed to be arranged in uninterrupted tandem arrays by restriction digest analysis and extended fiber FISH, and therefore DXZ4 encompasses 36–288 kb of Xq23. Transmission of DXZ4 through three generations in three families displayed a high degree of meiotic instability (8.3%), consistent with other macrosatellite arrays, further highlighting the unstable nature of these sequences in the human genome. Subcloning and sequencing of complete DXZ4 monomers identified numerous single nucleotide polymorphisms and alleles for the three microsatellite repeats located within each monomer. Pairwise comparisons of DXZ4 monomer sequences revealed that repeat units from an array are more similar to one another than those originating from different arrays. RNA fluorescence *in situ* hybridization revealed significant variation in DXZ4 expression both within and between cell lines. DXZ4 transcripts could be detected originiating from both the active and inactive X chromosome. Expression levels of DXZ4 varied significantly between males, but did not relate to the size of the array, nor did inheritance of the same array result in similar expression levels. Collectively, these studies provide considerable insight into the polymorphic nature of DXZ4, further highlighting the instability and variation potential of macrosatellites in the human genome.

## Introduction

At least half of the human genome is composed of repetitive DNA [1], including transposable elements, segmental duplications and tandem repeat DNA. Among the tandem repeats, relatively little is known about the role of macrosatellite arrays in the genome, many of which have yet to be described in detail [2].

Macrosatellites consist of repeat units ranging from 1–12 kb that are arranged in tandem. The number of repeat units is polymorphic in the general population, and an array can be composed of only a few to over one hundred repeat units, and therefore can encompass large genomic intervals. Most macrosatellite arrays are specific to one or two chromosomal locations in the genome, and only a small number have been confirmed and characterized to some extent [2]–[9]. Most is known about D4Z4, a tandem array of 1–100 3.3 kb repeat units located on chromosomes 4q35 and 10q26 [10], [11]. Contraction in the size of the 4q35 array to fewer than 10 repeat units is associated with the onset of fascioscapulohumeral muscular dystrophy (FSHD) [9], the third most common inherited form of muscular dystrophy [12]. More recently a possible link was made between the chromosome 5p15 TAF11-like array [3], [7] and schizophrenia, whereupon small arrays co-segregated with disease onset in four families [3]. However, despite links between these unusual DNA elements and disease, the role of many macrosatellites in the human genome remains unexplored.

**Figure 1:**
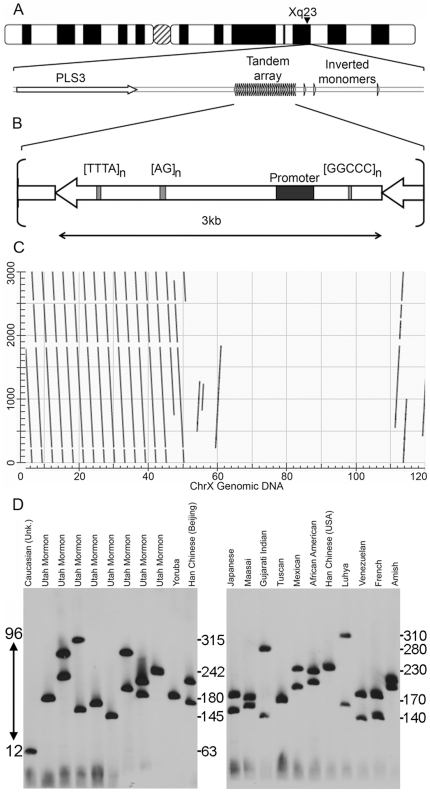
Organization and variation of DXZ4. (A) Ideogram of the human X chromosome showing the location of DXZ4 at Xq23. Beneath this is a schematic representation of the region immediately surrounding DXZ4. The macrosatellite array and distal inverted monomers are represented by the arrow heads. The nearest gene *PLS3* is indicated proximal to the array. (B) Representation of a single 3.0 kb DXZ4 monomer defined by *Hind*III. The internal microsatellite repeats are indicated as is the DXZ4 promoter region. (C) Predicted higher-order organization of the array as revealed by dot-plot analysis. A single 3.0 kb monomer sequence is on the y-axis, whereas the 120 kb genomic interval containing the array and inverted monomers is on the x-axis. The 120 kb sequence is located at 114.9 Mb on the human X chromosome (coordinates according to build hg19). Dot-plot generated using NCBI Blast, and the output image labeled in Adobe Photoshop CS2. (D) Copy number variation of DXZ4. Southern blot analysis of *Xba*I cut DNA from 22 unrelated individuals separated by pulsed field gel electrophoresis and hybridized with a DXZ4 probe. The ethnicity of the individuals used is indicated at the top. Size in kb is given to the right of each blot. The numbers given to the left of the blots with the double-headed arrow indicates the range of inferred DXZ4 copy number, with 12 in the smallest array and approximately 96 in the largest array.

Our interest in macrosatellite repeats came about through examination of chromatin organization on the human inactive X chromosome (Xi). X chromosome inactivation (XCI) is the mammalian form of dosage compensation [13], that balances levels of X-linked gene expression between the sexes by shutting down most transcription from one of the two female X chromosomes [14]. Gene silencing is achieved by repackaging the chosen Xi into facultative heterochromatin early in development. Chromatin of the Xi is composed of at least two types of heterochromatin that occupy distinct regions of the chromosome [15], [16]. Outside of the pseudoautosomal region, an area of the X chromosome shared with the Y that is not subject to dosage compensation [14], euchromatic markers are absent from the Xi, with the notable exception of DXZ4 [17], appearing as a euchromatic island embedded within the territory of the Xi [18]. DXZ4 is located exclusively on the X chromosome [5] at Xq23 approximately 74.5 kb distal to the plastin 3 gene (*PLS3*), and 296.5 kb proximal to the angiotensin II receptor (*AGTR2*)(Figure 1A). DXZ4 is composed of a 3.0 kb repeat unit arranged in tandem as many as 100 times [5]. The repeat monomer has a 62% GC content and over 180 CpG dinucleotides, and therefore is an extensive CpG island (CGI). With the exception of three internal microsatellite repeats (Figure 1B), that account for less than 5% of a monomer sequence, the remaining DNA sequence is unique. In contrast to other X-linked CGI's [19], [20], CpG dinucleotides on the active X chromosome (Xa) and on the male X were found to be methylated, whereas the DXZ4 array on the Xi was largely hypomethylated [5]. We confirmed these observations and showed that DXZ4 on the Xa is packaged into constitutive heterochromatin characterized by histone H3 lysine-9 trimethylation (H3K9me3), whereas DXZ4 on the Xi was packaged into euchromatin and bound by the epigenetic organizer protein CTCF [4]. Somewhat unexpectedly, DXZ4 on the Xa is expressed, despite being packaged into heterochromatin. Expression originates from a bi-directional promoter located within each monomer [4]. DXZ4 expression was also detected from the Xi. The purpose of DXZ4 transcription remains unclear, as does the extent of expression from either X chromosome, although it is tempting to speculate that expression of DXZ4 influences its chromatin packaging.

Here we report our findings on four aspects of DXZ4 variation: tandem repeat copy number variation, array instability, monomer sequence polymorphism and differences in array expression.

## Results

### Characterization and copy number variation of the DXZ4 macrosatellite

Assembly of large tandem repeat DNA sequence such as DXZ4 is particularly challenging for computer sequence alignment programs. For example, two or more sequences that share 100% sequence identity may reside adjacent to one another within an array *in vivo*, but would be aligned on top of one another *in silico*. A comparison of a single DXZ4 monomer sequence (Accession Number HQ659112) against the assembled human genome sequence (hg19) using the UCSC genome browser (http://genome.ucsc.edu/), reveals approximately fourteen ∼3 kb DXZ4 monomers covering 50 kb, arranged in tandem centered at 115 Mb on the X chromosome. In addition, two partial and one complete monomer reside in an inverted orientation relative to the main array within the immediate distal 70 kb sequence (Figure 1C). These likely account for the DXZ4 hybridizing invariant fragments described previously [5].

In the same report [5], DNA from 17 unrelated individuals digested with *Eco*RI and separated by pulsed field gel electrophoresis (PFGE) revealed hybridizing DXZ4 *Eco*RI fragments of between 150–300 kb by Southern analysis. This translates into arrays composed of between 50 to 100 monomers. We extended this analysis to an additional 22 unrelated individuals of diverse ethnicity. Agarose embedded genomic DNA was digested with *Xba*I (a restriction endonuclease for which there are no recognition sites within DXZ4) and were then separated by PFGE before transfer to nylon membrane by Southern blotting. As expected, hybridization of the blots with a DXZ4 probe identified two hybridizing signals in female samples and one in males (Figure 1D). An identical pattern was obtained when DNA was digested with *Pvu*II (Figure S1), another restriction endonuclease for which no recognition sequences are present in DXZ4. Among the 36 alleles, fragment sizes ranged from 63 to 315 kb with an average size of 198 kb and a median of 189 kb. Based on the assembled genomic sequence flanking the array, the closest *Xba*I site is approximately 0.7 kb proximal and 25.3 kb distal. Adjusting for this additional non-array DNA sequence, we can infer between 12 and 96 individual 3 kb DXZ4 monomers, with an average of 57 and median of 54 monomers per DXZ4 array. Therefore, DXZ4 represented in the hg19 genome sequence build is within the size range of DXZ4 arrays observed *in vivo*, albeit on the smaller size.

### Confirmation of tandem arrangement of DXZ4

Next we sought to confirm that DXZ4 is indeed a tandem array of individual 3 kb monomers arranged in a head-to-tail orientation. In order to do this we used two complementary approaches; restriction endonuclease digest analysis of a DXZ4 bacterial artificial chromosome (BAC) clone, and extended DNA fiber fluorescence *in situ* hybridization (FISH).

**Figure 2:**
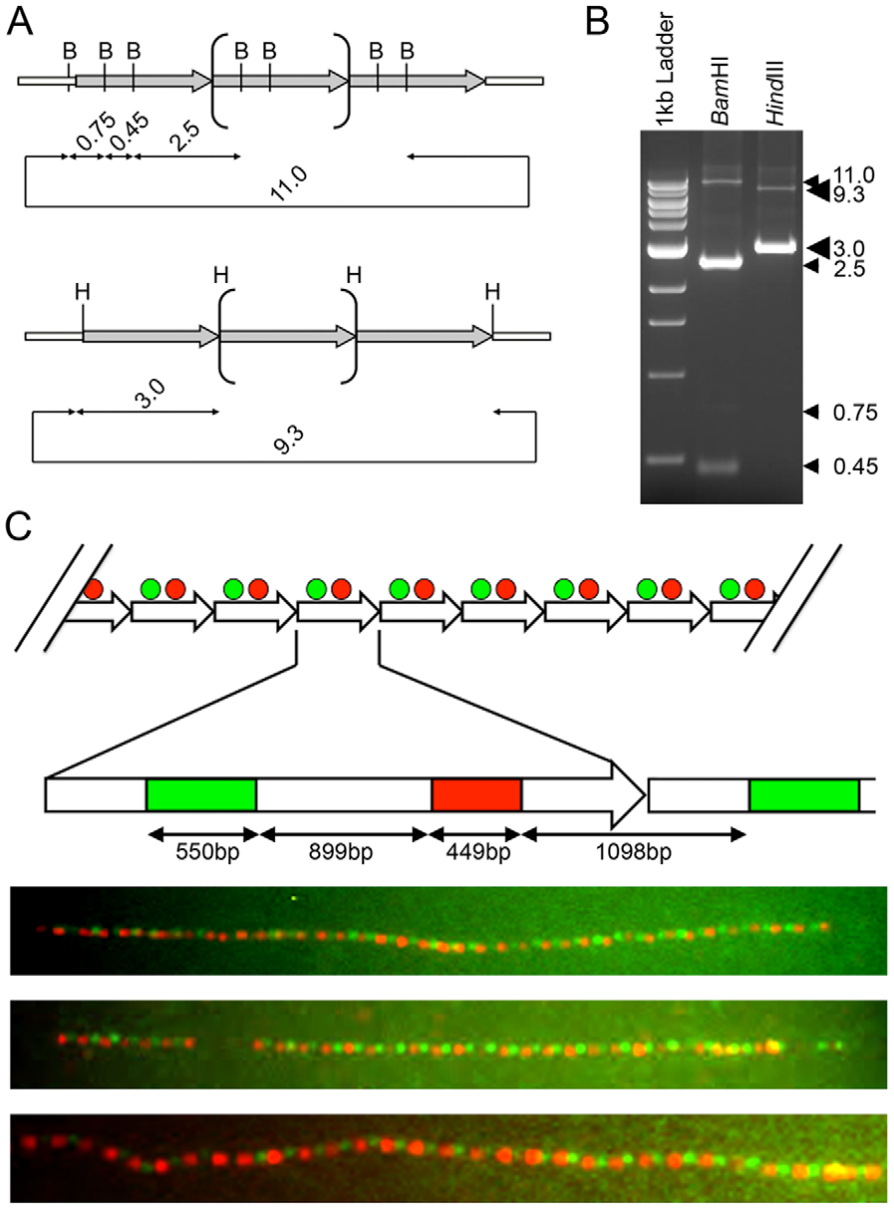
Confirmation of tandem arrangement for DXZ4. (A) Predicted restriction endonuclease map for DXZ4 BAC clone 2272M5 using *Bam*HI, B top, or *Hind*III, H bottom. Predicted fragment sizes given are in kb. The grey right facing arrows represent single 3 kb DXZ4 monomers. The central bracketed monomer represents all other tandem arranged monomers in the BAC. The large looped arrow (11.0 or 9.3) represents the pBeloBAC11 vector backbone. (B) Ethidium bromide stained agarose gel showing restriction fragments obtained from BAC 2272M5 when digested with either *Bam*HI or *Hind*III. Fragment sizes are given to the right. (C) Tandem arrangement of DXZ4 *in vivo* as determined by extended fiber FISH. At the top is a predicted schematic for DXZ4 tandem arrangement, with each right facing arrow representing a 3.0 kb DXZ4 monomer. The alternating red and green circles represent probe locations. Beneath this is a representation of a single DXZ4 monomer indicating the location of the two probes used for fiber FISH of 550 bp (Green) and 449 bp (Red) separated by 899 bp or 1098 bp for the adjacent monomer. At the bottom are examples of merged Red and Green fluorescent images of extended DNA fiber hybridizations. Yellow signals indicate overlapping red and green probes in regions where fibers are not stretched to the same extent as the rest of the fiber.

In order to identify BAC clones that matched DXZ4 at both ends, the DNA sequence of a DXZ4 monomer was compared to entries in the Genome Survey Sequence database using BLAST (http://blast.ncbi.nlm.nih.gov/Blast.cgi). Several clones were identified including clone 2272M5 from the human genomic sperm CITB BAC library D (Accession number AQ745776). The BAC clone was obtained and DNA isolated. Given that the BAC clone insert matches DXZ4 sequence at both ends, uninterrupted tandem arrangement of DXZ4 monomers should result in the generation of a predictable pattern of restriction fragments (Figure 2A). *Bam*HI cuts twice per monomer and once in the BAC vector (pBeloBAC11). *Hind*III cuts once per monomer and is the cloning site used for generation of this library [21]. Restriction endonuclease digestion of BAC 2272M5 was consistent with an uninterrupted tandem array of DXZ4 monomers (Figure 2B). The higher intensity of the 2.5 kb *Bam*HI and 3.0 kb *Hind*III fragment relative to the vector backbone fragment indicated that the BAC contained several DXZ4 monomers. To confirm this, the BAC clone was digested with four different restriction endonucleases that have recognition sites within the vector backbone but not within DXZ4, and the cut DNA was separated by PFGE. For all four digests the resulting fragment was greater than 100 kb (Figure S2). The *Not*I digest excises the insert from the 7.4 kb vector backbone and therefore the approximate insert size for BAC clone 2272M5 is 110 kb, indicating the presence of as many as 37 tandem arranged DXZ4 monomers.

In order to confirm tandem arrangement of DXZ4 *in vivo*, we used extended DNA fiber FISH. A 449 bp and 550 bp region from a single DXZ4 monomer separated by at least 899 bp were PCR amplified and ligated into the TA-cloning vector pCR2.1 (Invitrogen). The two cloned fragments were then labeled with different fluorophores and used for FISH. A tandem arrangement of DXZ4 would result in an alternating red-green signal, as was observed (Figure 2C). Such an approach consistently resulted in arrays of 30 or more tandem DXZ4 monomers. Occasionally, the red-green alternating pattern would be interrupted by a gap (See Figure 2C, middle sample). DNA fibers are prone to breakage, and therefore it is more likely that such a gap represents a break in the DNA fiber and not an interruption in the array by non-DXZ4 DNA. In support of this statement, gaps were observed alongside uninterrupted tandem array patterns in fiber preparations from the same male samples (Data not shown). Given that males have only one DXZ4 allele, the gap represents a fiber break. On a technical note, these data indicate that resolving sequences less than 1 kb apart is feasible by fiber FISH, which is at the theoretical lower limit of resolution of light microscopy [22].

### Monomer repeat variation

In addition to exploring variation in the number of DXZ4 monomers in an array, we also investigated sequence variation between different monomers. The strategy that we chose was to subclone individual DXZ4 monomers from BAC clone 2272M5 using *Hind*III. By using this one BAC source we ensured that all monomers were derived from a single DXZ4 array. Therefore we could compare monomers within an array and against monomers from other arrays. Subclones were then sequenced using five oligonucleotide primers from each strand (See PCR Primers S1). Complete sequences were assembled using Sequencher 4.10.1, and 18 monomers were identified that showed sequence variation relative to one another (Table 1). According to the characterization of the BAC described above, as many as 37 monomers are present in 2272M5. We did not sequence sufficient number of subclones to be confident that all variants within the BAC had been identified. However, our identification of at least 18 polymorphic DXZ4 monomers indicates that almost half of the repeat units within the BAC are different, accounting for greater than 50 kb of the insert.

**Table 1:** Summary of DXZ4 monomer variation.

Subclone	SNPs	(GGGCC)	(CT)	(TAAA)
35	Reference	5	16	13
1	A-G^207^, C-G^364^, T-C^828^	2	12	8
3	-	5	16	10
8	-	5	16	8
15	C-T^26^, G-C^1890^, C-A^1940^	5	16	12
16	G-C^1890^, del T^1889^	5	15	10
18	G-C^1890^, C-A^1940^	5	16	10
22	A-G^207^, C-G^364^	3	11	10
23	-	5	14	9
31	G-C^1890^, C-A^1940^, C-G^2636^	5	14	10
40	G-C^1890^	5	16	10
46	-	5	14	10
47	A-G^207^, C-G^364^, T-C^828^	2	16	12
59	del T^2286^, del T^2309^	5	11	10
69	A-G^207^, C-G^364^, T-C^828^, G-C^1890^, G-C^2007^, T-A^2670^	2	9	11
70	G-C^1890^	5	15	10
87	G-C^1890^	5	13	10
88	G-C^1890^, C-A^1940^	5	16	9

Table showing sequence variation for the 18 DXZ4 monomer subclones isolated from BAC clone 2272M5. SNP coordinates are based on the reference sequence of subclone 35. Absent bases are given the prefix “del”. Copy number of the pentameric (GGGCC), tetrameric (TAAA) and dimeric (CT) microsatellites are indicated. Accession numbers for monomers listed above are as follows: DXZ4-1 - HQ659103; DXZ4-3 - HQ659104; DXZ4-8 - HQ659105; DXZ4-15 - HQ659106; DXZ4-16 - HQ659107; DXZ4-18 - HQ659108; DXZ4-22 - HQ659109; DXZ4-23 - HQ659110; DXZ4-31 - HQ659111; DXZ4-35 - HQ659140; DXZ4-40 - HQ659113; DXZ4-46 - HQ659114; DXZ4-47 - HQ659115; DXZ4-59 - HQ659116; DXZ4-69 - HQ659117; DXZ4-70 - HQ659118; DXZ4-87 - HQ659119; DXZ4-88 - HQ659120.

The 18 unique monomers shared 99% or greater sequence identity according to pairwise alignments using BLAST (http://blast.ncbi.nlm.nih.gov/Blast.cgi). Most variation was accounted for by polymorphism in copy number of repeat units in the three internal microsatellite repeats. The [GGGCC] repeat ranged from 2 to 5 tandem copies, the [CT] repeat ranged from 9 to 16 tandem copies, and the [TAAA] repeat ranged from 8 to 13 tandem copies (Table 1). DXZ4 monomers within the tandem array in hg19 all had 5 tandem copies of the [GGGCC] repeat, between 11 and 19 tandem copies of the [CT] repeat and 8 to 12 tandem copies of the [TAAA] repeat (Table S1). The DXZ4 monomer sequenced by Giacalone and colleagues [5] was consistent with 5 [GGGCC], 18 [CT] and 8 [TAAA] (Table S2). The largest [GGGCC] repeat of 8 tandem copies and [CT] repeat of 20 tandem copies were identified in monomer sequences from a fosmid library derived from a Japanese individual (accession number AC212298.1) [23].

**Figure 3:**
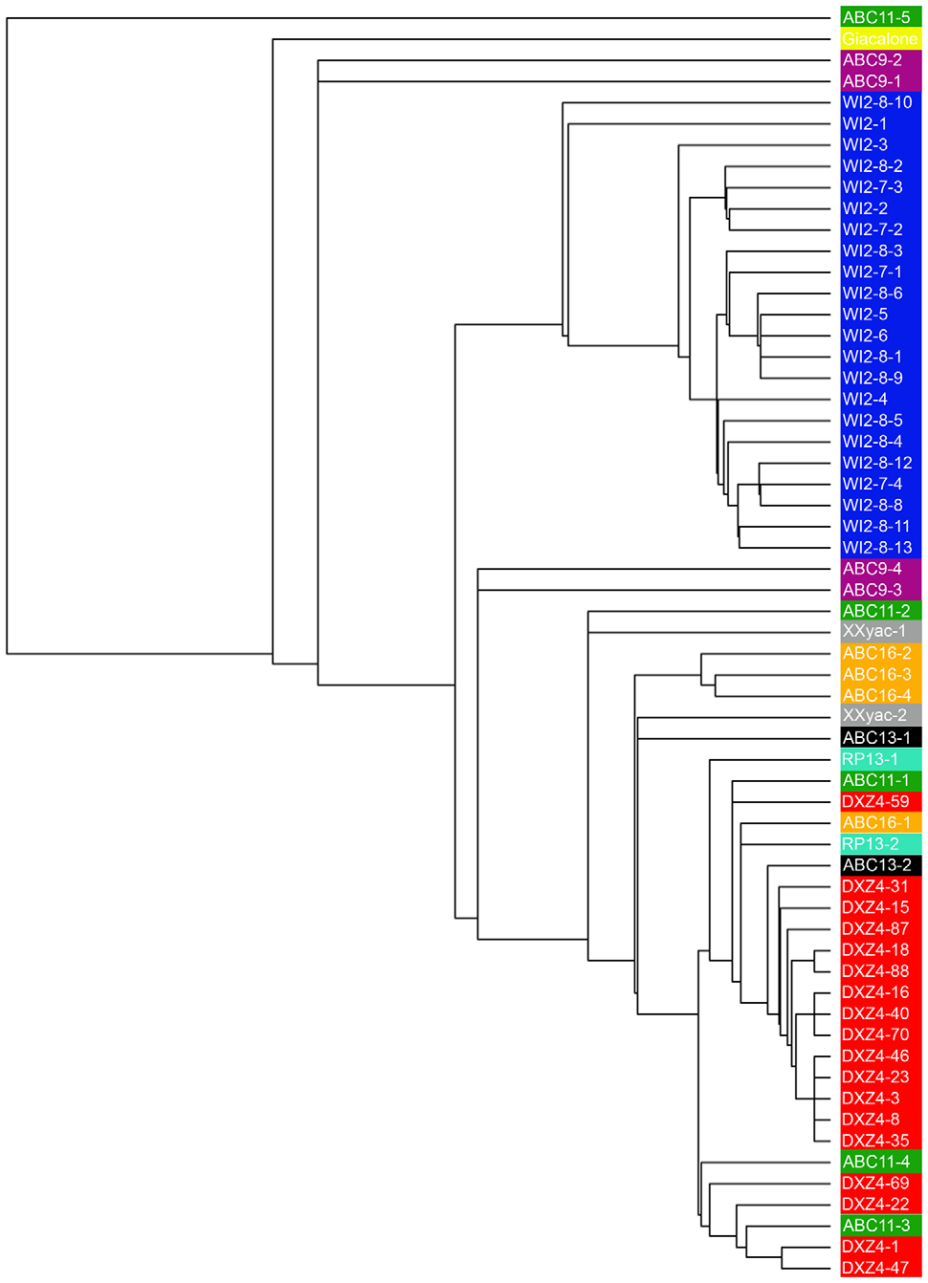
Relationship of DXZ4 monomer sequence within and between individuals. Cladogram of 60 complete DXZ4 monomer DNA sequences. Tree image generated using MUSCLE version 3.8 [37]. Color highlights added in Adobe Photoshop CS (Ver.8.0). Sequences labeled DXZ4-1 through 88 and highlighted in red represent sequences derived from BAC clone 2272M5 (this manuscript). Sequences labeled WI2 and highlighted in blue represent sequences taken from clones from the WIBR-2 human fosmid library. Sequence accession numbers include WI2-7 AC196704.1, WI2 AC193162 and WI2-8 CR753863.9. Sequences labeled ABC and colored green, mauve, orange and black are derived from fosmid libraries from four individuals of different ethnicities [23]. Sequence accession numbers include: ABC9 AC212298.1, ABC11 AC236928.2, ABC13 AC226798 and ABC16 AC238719.3. The sequence highlighted in yellow annotated Giacalone is taken from accession number S60754 [5]. Sequences labeled XXyac highlighted in grey are derived from XXyac-74A3 (BX546444.14). RP13 sequences derived from accession number AL392170.7.

In addition to the microsatellite polymorphism, novel single nucleotide polymorphisms (SNPs) were identified in monomers from 2272M5 (Table 1), as well as 18 additional unique SNPs and 2 insertions in monomers from hg19 (Table S1) and 11 unique SNPs and two insertions in the Giacalone monomer (Table S2). The sequence of a complete DXZ4 monomer was used to search the public databases and identify sequenced clones containing at least two complete DXZ4 monomers. These sequences were collected and used along with the 2272M5 subclone sequences and the Giacalone sequence to compare the sequence relationship between all of the monomers. We found that DXZ4 monomers originating from the same tandem array shared higher DNA sequence identity with one another than with monomers from a different array. All monomers originating from human fosmid library WIBR-2 (prefixed WI2) clustered together (Figure 3). Likewise, monomers from BAC 2272M5 clustered together and were more similar to one another than to any of the WI2 monomers. Two of the monomer sequences extracted from the public databases were clear outliers showing the least similarity to the other 58 monomers (Figure 3). The first is a single monomer originating from a fosmid library derived from a Han Chinese individual (ABC11-5). This sequence differed from others in this individual mostly by 11 base insertions and 7 base deletion polymorphisms. The second outlier is the Giacalone monomer for which no other monomer sequences from this individual are available.

### Stability of DXZ4

**Figure 4:**
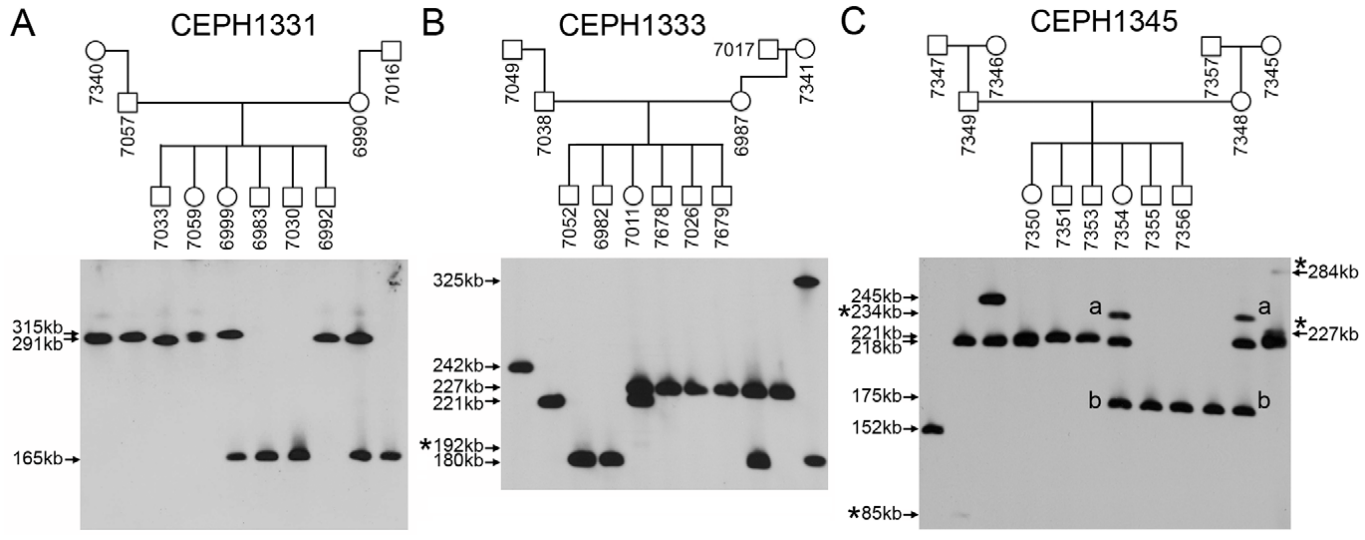
DXZ4 inheritance and stability. Inheritance of DXZ4 through three generations in three independent CEPH Utah pedigrees. (A) CEPH-1331, (B) CEPH-1333 and (C) CEPH-1345. Members of each family are indicated above the blot in the pedigrees and the members are given the Coriell GM0- ID for each member of the family. Hybridizing fragments are given to the left and right sides of the blots. The asterisks indicate alleles of altered size.

The range in allele sizes observed for DXZ4 (Figure 1D) indicate that like other macrosatellite arrays [2], [3], [6], [7], [9], [24] DXZ4 is prone to contraction or expansion. In order to investigate DXZ4 stability, we examined DXZ4 transmission through three generations using three independent Jean Dausset-Centre d'Etude du Polymorphisme Humain (CEPH) families (Figure 4). As expected, DXZ4 inheritance conforms to sex linkage with all male offspring only inheriting an allele from their mother. Consistent with the data from the variation panels (Figure 1D), alleles ranged from 165 to 325 kb that translate into arrays of 46 to 100 DXZ4 monomers. Members of CEPH family 1331 (Figure 4A) showed no evidence of meiotic or mitotic instability. DXZ4 alleles in CEPH family 1333 also showed no signs of meiotic instability. However, a weak allele of 192 kb was consistently observed for individual 7011 (Figure 4B), indicative of mitotic instability. It is not possible to determine from which allele the smaller array is derived due to the fact that it represents instability in a very small number of cells. In CEPH family 1345 (Figure 4C), a similar situation was observed for the father 7349, whereupon a weak 85 kb band was observed alongside the inherited 218 kb allele. Neither daughter (7350 and 7354) nor his mother (7346) show the presence of this additional band confirming that this is mitotic instability restricted to the fathers somatic cells. Evidence of meiotic and mitotic instability was observed on the maternal side of the family. The grandmother (7345) has a single allele of 221 kb as well as two additional weaker hybridizing bands of 227 and 284 kb (Figure 4C). The 221 kb allele is stably inherited through the mother (7348) to two sons (7351 and 7353) and two daughters (7350 & 7354). However, the mother shows the appearance of a new allele that is characterized by a hybridizing band of 234 kb (labeled “a”) that is indicative of meiotic instability. In addition, the mother has inherited a 175 kb allele (labeled “b”) from her father (7357). The new “a” band most likely has arisen from meiotic instability via the grandfather, because one daughter (7354) has stably inherited both “a” and “b” while also inheriting the 218 kb array from her father. Unusually, two sons (7355 and 7356) do not show inheritance of the “a” hybridizing band, but only the “b” fragment. One possible explanation for this observation could be experimental error whereby cell line 7348 is contaminated with DNA/cells from an unrelated individual (explaining the additional band), and 7354 is a duplicate of the contaminated 7348. In order to ensure that this was not the case, the same blot was hybridized with probes to one other X-linked tandem repeat and two autosomal macrosatellites. In all three cases, both individuals showed alleles consistent with Mendelian inheritance and the complete absence of any additional alleles (Data not shown). Furthermore, agarose embedded plugs for the same family cut with *Pvu*II, separated by PFGE and Southern blotted, showed the same pattern of bands when hybridized with a DXZ4 probe (Figure S3), indicating that the change in fragment sizes are not simply the result of gain or loss of an *Xba*I site in the array.

### Expression analysis of DXZ4

Previously we have shown that DXZ4 is expressed from a bi-directional promoter located within each monomer [4]. Our interpretation of strand specific reverse transcription PCR (RT-PCR) and Northern blot analysis was that from the Xa allele, DXZ4 produces a long abundant RNA from one strand and several short RNA's from the opposite strand. In contrast, the DXZ4 array on the Xi produces the same two RNA species as well as a weak longer RNA from the opposite strand. To investigate the expression of DXZ4 further we performed direct-labeled RNA FISH with a DXZ4 probe alongside a direct-labeled XIST probe in order to view transcripts without amplification and to assign expression as originating from the Xa or Xi.

**Figure 5:**
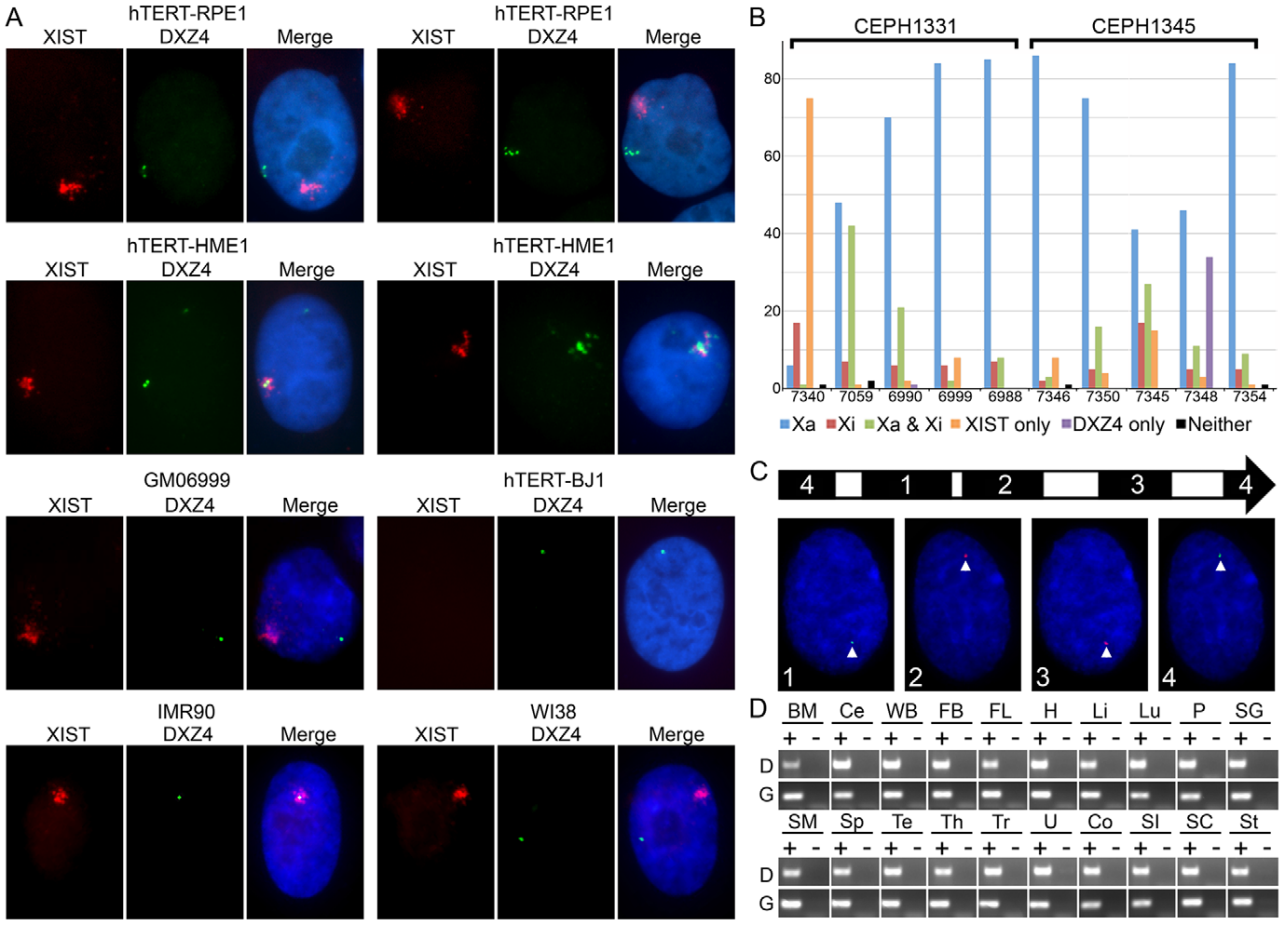
Expression of DXZ4. (A) Examples showing the distribution of DXZ4 RNA versus XIST RNA in various 46,XX cells and a 46,XY cell line. The cell identity is indicated above each panel of three images. XIST RNA is shown in red, DXZ4 in green and nuclei are stained with DAPI (blue) in the merged image. (B) Expression of DXZ4 from the Xa and Xi in ten independent female lymphoblast cell lines given on the x-axis. Number of nuclei are given on the y-axis as percent. One hundred nuclei were scored for each cell line. (C) Detection of distinct regions of DXZ4 RNA by RNA FISH. The right facing arrow represents a single DXZ4 monomer with the regions highlighted in black (1–4) indicating the location of the probes used. Beneath this are examples of RNA FISH signals for each probe merged with DAPI. (D) RT-PCR analysis of DXZ4 using cDNA generated from total RNA isolated from 20 different human tissues. The tissues are listed above each agarose gel image, with a “+” indicating cDNA with reverse transcriptase and “−” indicating the no reverse transcriptase control. The top row indicates DXZ4 (labeled “D”) whereas the lower lane indicates GAPDH (labeled “G”). BM – bone marrow; Ce – cerebellum; WB – whole brain; FB – fetal brain; FL – fetal liver; H – heart; Li – liver; Lu – lung; P – prostate; SG – salivary gland; SM – skeletal muscle; Sp – spleen; Te – testis; Th – thymus; Tr – trachea; U – uterus; Co- colon; SI – small intesitine; SC – spinal cord; St – stomach.

Expression was initially assessed in two female and one male diploid telomerase immortalized cell lines (hTERT), two diploid female primary fibroblast cultures and one EBV transformed diploid female lymphoblast cell line. Representative examples of the results are shown in Figure 5A. For hTERT-RPE1, DXZ4 was clear and distinct from the XIST signal in 93% of nuclei (n = 200). Only 3% of nuclei had a single DXZ4 signal inseparable from XIST, 1% showed expression from both the Xa and Xi and 3% of nuclei did not show any DXZ4 signal. On occasion the DXZ4 signal was extensive and approaching the size of the XIST signal (Figure 5A, top panel right set of three). For hTERT-HME1, only 21% of nuclei (n = 100) had DXZ4 signals distinct from XIST, whereas 58% showed expression from both the Xa and Xi (Figure 5A, left panel set) and 14% showed expression from the Xi only. As with RPE1, DXZ4 expression occasionally was extensive with a signal comparable in size to the XIST territory (Figure 5A, right panel set). For both RPE1 and HME1, the extensive DXZ4 signals from the Xi showed minimal overlap with the XIST signal. In the lymphoblast cell line GM06999, DXZ4 was primarily detected from the Xa (84% of nuclei, n = 50) with only 6% of nuclei showing DXZ4 at the Xi only and 2% showing DXZ4 from both the Xi and Xa. In the primary fibroblast cultures, DXZ4 was almost always expressed from the Xi in IMR90 and from the Xa in WI38 (Figure 5A, bottom panels). A third female primary fibroblast culture showed expression of DXZ4 from the Xa in 75% of nuclei (data not shown). Collectively, these data indicate that expression of DXZ4 is variable and can originate from the Xa, Xi or both within and between cell types.

Next we sought to investigate the expression of DXZ4 from the Xi and Xa in 10 different female lymphoblast cell lines by RNA FISH with direct labeled probes; five from CEPH family 1331 and five from family 1345. DXZ4 expression was readily detected in all females (Figure 5B). The paternal grandmother in family 1331 showed the least number of cells expressing DXZ4, with most cells XIST positive only. In those cells expressing DXZ4, some were expressed from the Xa only and others from the Xi only with few cells expressing both. Granddaughter 7059, who inherited one of her paternal grandmothers DXZ4 alleles showed DXZ4 expression in almost all cells, with over 40% showing DXZ4 from the Xa alone and another 40% showing DXZ4 expression from both the Xa and Xi. Mother 6990 and daughters 6999 and 6988 also showed DXZ4 expression in almost all cells, with most expression coming from the Xa. In CEPH family 1345, paternal grandmother 7346 and granddaughter 7350, who inherited one of her paternal grandmothers alleles, show most cells expressing DXZ4 from the Xa. Forty percent of cells in maternal grandmother 7345 expressed DXZ4 from the Xa. Of the remaining approximately 60% of cells, DXZ4 expression was observed from the Xi alone, Xa and Xi or not at all with similar frequency. Of note, this individual demonstrated both meiotic and mitotic instability of DXZ4 (Figure 4C). Her daughter, 7348, who inherited a new DXZ4 allele as a result of her fathers meiotic instability, still showed over 40% of cells expressing DXZ4 from the Xa only, but in addition over 30% of cells showed DXZ4 expression in the absence of XIST. One possible explanation could be that these cells have lost the Xi. However, allele intensities observed by PFGE Southern hybridization are almost identical to her daughter (compare bands “a” and “b” for 7348 and 7354, Figure 4C). Therefore it is more likely that XIST was not being expressed from the Xi in these cells. Interestingly, daughter 7354 who inherited the same allele showed DXZ4 expression from the Xa in over 80% of cells, with a similar DXZ4 expression profile to several other related and unrelated females.

Previously, we had shown that all regions of DXZ4 could be detected by RT-PCR [4]. However, these data represented end-point analysis of PCR on agarose gels and not quantitative RT-PCR (QRT-PCR), and therefore different regions of DXZ4 could be expressed at different levels. The high GC content (∼62%) of DXZ4 makes QRT-PCR at some regions of DXZ4 challenging. Therefore we performed RNA FISH with short direct-labeled probes to four regions spanning most of a DXZ4 monomer. DXZ4 RNA was readily detected with all four probes at comparable intensities, confirming earlier RT-PCR data that all of DXZ4 is likely expressed (Figure 5C).

Finally, we examined expression of DXZ4 in a panel of complementary DNA (cDNA) samples prepared from 20 different human tissues. RT-PCR analysis showed robust expression of DXZ4 from all sources (Figure 5D) indicating that expression of DXZ4 is ubiquitous.

### DXZ4 expression does not correlate with array size or allele inheritance

**Figure 6:**
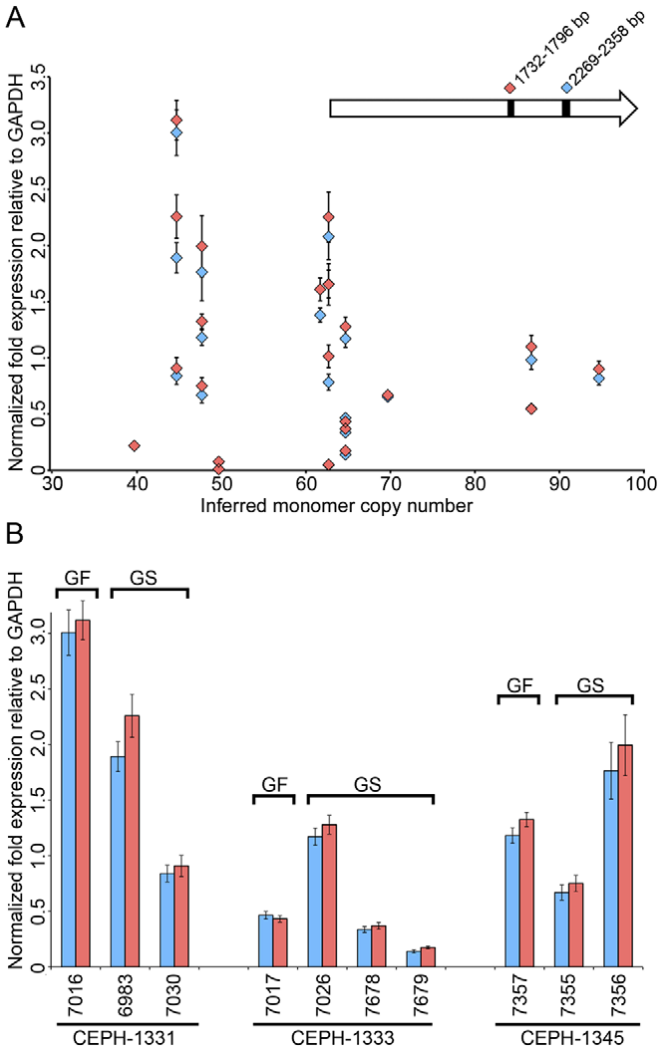
Lack of correlation between array size, expression and inheritance. (A) Graph showing normalized expression levels of DXZ4 relative to GAPDH (y-axis) plotted against the inferred monomer copy number of DXZ4 (x-axis) for 22 different males as determined by PFGE. The right facing arrow represents a single DXZ4 monomer with the location of the regions amplified indicated. The plotted data indicates qRT-PCR for each of the two regions (pink diamond v blue diamond) from triplicate amplifications. (B) Expression levels of DXZ4 in maternal grandfathers (GF) plotted alongside grandsons (GS) who inherited their maternal grandfathers DXZ4 array. Data shown for three independent CEPH families.

Like DXZ4 [4], multiple RNA species originate from the autosomal macrosatellite D4Z4 [25]. In myoblasts from one FSHD patient, higher levels of D4Z4 derived RNA could be detected [26], which may reflect stabilized transcripts originating from the most distal edge of the contracted array [27]. Therefore, we explored the possibility that expression of DXZ4 might be associated with the size of the array. In order to do this, we performed QRT-PCR to two regions of DXZ4 on cDNA prepared from male samples. Because males are hemizygous for DXZ4, the size of the array as determined by PFGE and Southern blot analysis (Figures 1 and 4) could be directly correlated to the level of DXZ4 transcript. A total of 22 males were examined, and their inferred monomer copy number was plotted against expression levels of DXZ4 (Figure 6A). On first examination of the resulting graph, it would appear that smaller arrays showed higher expression of DXZ4. However, smaller arrays also showed the lowest levels of DXZ4 expression. Therefore, we conclude that DXZ4 expression levels are highly variable, and from this limited sample size expression does not directly correlate with array size. While the range of expression is greater for smaller arrays, more male samples with large arrays would be needed to determine if this is significant.

Next we sought to examine if inherited DXZ4 alleles showed comparable expression levels. Once again, to ensure expression originated from a single allele we restricted our analysis to males. Due to X-linkage we also restricted our analysis to sons inheriting DXZ4 from their maternal grandfather. As with the size versus expression analysis described above, DXZ4 expression was variable (Figure 6B), and inheritance of an array did not result in comparable expression levels from the array.

## Discussion

Exploring variation in the human genome is essential to begin to understand how polymorphism impacts gene expression, phenotypic variance and disease susceptibility. Here we report on variation of the X-linked macrosatellite DXZ4.

In this study, we confirm that DXZ4 is a polymorphic uninterrupted tandem array, and extend the range of observed alleles to between 12 and 100 head-to-tail 3 kb repeat units. This variability is comparable to that described for other macrosatellites [2], [3], [6], [7], [9], [24], with the smallest DXZ4 allele approaching the pathogenic D4Z4 array size associated with FSHD [8], [9]. Two of twenty-four parent to offspring transmissions show evidence of meiotic instability (8.3%). This high frequency of mutability is comparable to that described for other macrosatellite arrays [6], [7] highlighting the germ line instability of these regions of the genome. Furthermore, this rate is similar to that reported for mini-satellite tandem repeats [28], [29], suggesting that the molecular mechanism through which repeat copy number is altered [30] is common and independent of repeat unit size.

In addition to monomer copy number variation, we report variation in both the internal microsatellite repeats, and numerous SNPs within the unique regions of a monomer. Our analyses indicate that DXZ4 monomers within one array are more similar to one another than they are to monomers in an array from a different individual. This suggests that mutations acquired in a monomer can spread through an array, most likely via complex gene conversion mechanisms as has been described for other satellite DNA [31].

Previously we have described several different RNA species originating from DXZ4, with expression from both the Xa and Xi [4]. Here we report considerable variation in the levels of expression of DXZ4 from the lone X chromosome in males. Expression levels do not appear to be associated with the size of the DXZ4 allele indicating that not all monomers within an array are expressed to the same extent. Furthermore, expression levels are not similar in individuals inheriting the same allele. It is possible that levels of DXZ4 expression are instead associated with the degree of monomer promoter methylation and extent of H3K9me3. However, such an analysis may have to be conducted using primary cells and/or white blood samples, as recent reports indicate changes in DNA methylation patterns in EBV-transformed lymphoblasts in culture [32], [33].

As with expression of DXZ4 from the Xa, DXZ4 expression from the Xi is variable, differing both between cell lines and from one cell to the next within the same line. Using direct RNA FISH, it is also clear that the level of expression of DXZ4 differs from cell to cell within the same cell line; with some cells showing little to no expression whereas others may show DXZ4 localized transcript patterns comparable in size to that of XIST RNA. It is important to note that large diffuse DXZ4 signals were not common, and were only observed in the hTERT and EBV transformed cell lines. However, primary cells were not extensively examined in this study and therefore no significance can be placed on the extent of the DXZ4 signal and cell immortalization. DXZ4 monomers on the Xi are packaged into both euchromatin and heterochromatin [4], and therefore the degree of expression of DXZ4 from the Xi might be associated with the proportion of the Xi array packaged into heterochromatin. Testing such a hypothesis would require isolation of clonal cell populations to ensure that the same array was always on the Xi. Previously, we have shown by strand specific RT-PCR that most DXZ4 transcript originates from one strand of DXZ4 with low levels of anti-sense transcript only observed in female samples [4]. One interpretation of these data is that anti-sense expression is originating from the Xi. The RNA FISH method used here does not distinguish between the sense and anti-sense DXZ4 transcripts. Therefore it is feasible that RNA FISH signals observed from the Xi might in fact be anti-sense transcript, whereas signals at the Xa could be sense transcript. Future analysis with strand-specific FISH probes will address this question.

Recently we described the characterization of four autosomal macrosatellite arrays [7]. Three of the arrays were primarily expressed in the testis, with some expression in the brain, suggesting that at least these three macrosatellites may be new cancer-testis loci (CT). The exception was the SST1 array that is expressed in all tissues examined. Here we show that DXZ4 is also expressed at comparable levels in all tissues, and therefore DXZ4 is not a CT loci, distinguishing DXZ4 from two other X-linked macrosatellite repeats CT47 [34] and the GAGE locus [35] that are members of the CT family. The significance of DXZ4 expression is unclear. Like SST1, DXZ4 shows no obvious protein coding function. It is possible that expression of DXZ4 is associated with chromatin organization of the array [4], a theory that we are actively pursuing.

The data we present here extends our knowledge base of DXZ4 and the biology of macrosatellite arrays, providing the basis for formulation of new hypotheses to explore the role of DXZ4 on the X chromosome.

## Materials and Methods

### Reverse Transcription PCR

Human tissue total RNA was obtained from Clontech (636643). Residual genomic DNA was removed by pre-treating the RNA with DNaseI (Invitrogen) for 20 minutes at room temperature, before heat inactivating the DNaseI at 70°C in the presence of 2.5 mM EDTA for 15 minutes. cDNA was prepared using 1 ug of total RNA with or without M-MLV reverse transcriptase (Invitrogen) according to the manufacturers instructions.

cDNA was amplified using Taq polymerase (NEB) with the following cycle: 95°C for 2 minutes, followed by 35 cycles of 95°C 20 seconds, 58°C 20 seconds, 72°C 30 seconds. The sequence of oligonucleotides used to amplify DXZ4 cDNA and the anticipated product size are given in the Supporting Information (PCR Primers S1).

QRT-PCR was performed on a CFX96 thermocycler and analyzed using the CFX Manager Software (Biorad). The sequence of oligonucleotides used to amplify DXZ4 cDNA are given in the Supporting Information (PCR Primers S1). Amplification used IQ SYBR Green Supermix (Biorad).

### Cell lines

Lymphoblastoid cell lines of CEPH family members and the individuals used in the variation panels were obtained from the Coriell Institute for Medical Research (www.coriell.org/). Cells were maintained according to Coriell recommendations. Culture media (RPMI), fetal bovine serum and supplements were all obtained from Invitrogen corp. Telomerase immortalized cell lines hTERT-RPE1 (C4000-1 46,XX retinal pigment epithelia), hTERT-HME1 (C4002-1, 46,XX breast epithelia) and hTERT-BJ1 (C4001-1, 46,XY foreskin fibroblast) were all obtained from Clontech and maintained as recommended by the supplier. Fetal lung fibroblast primary cells IMR-90 (CCL-186, 46,XX) and WI-38 (CCL-75, 46,XX) were obtained from the American Type Tissue Culture Collection (ATCC), and maintained according to supplied instructions.

### Extended DNA Fibers & FISH

Lymphoblast cells were pelleted, washed with 1× PBS before resuspension in 0.075 M KCl and incubating at 37°C for 10 minutes. Cells were pelleted and resuspended in 3∶1 fixative (3 parts methanol, 1 part acetic acid). Cells were pelleted three more times, resuspending each time in 3∶1 fix. Fixed cells were either stored at −20°C or used immediately for fiber preparation. Cells in 3∶1 fix were applied to the raised end of a poly-L lysine coated microscope slide propped up on a paper towel. Using a cover glass, the cells were gently drawn down the length of the slide, dragging only the liquid, not touching the glass slide. Cells were air dried for 5 minutes before immersing in 3∶1 fix for 5 minutes. Fibers were dehydrated through 70% and 100% ethanol for 2 minutes each before air-drying. Fibers were denatured for 5 minutes in 70% formamide, 2× SSC at 75°C before dehydrating for 3 minutes each in cold 70% and 100% ethanol.

FISH probes consisted of 449 bp or 550 bp pCR2.1 cloned PCR fragments of DXZ4 that are approximately 900–1100 bp apart in a single monomer (PCR Primers S1). Probes were labeled with Spectrum Orange or Spectrum Green by Nick Translation according to the manufacturers instructions (Abbot Molecular), followed by ethanol precipitation and resuspension in 0.1 ml of Hybrisol VII (MP Biomedicals). A 1∶1 mix of the two probes were denatured at 75°C for 4 minutes, quenched on ice then applied directly to the slide, covered with cover glass, sealed with rubber cement and hybridized for 16 hours at 37°C. Slides were washed at 37°C twice in 50% formamide, 2× SSC for 8 minutes each, then once in 2× SSC for 8 minutes before adding ProLong Gold antifade containing 4′, 6-Diamino-2-phenylindole dihydrochloride (DAPI)(Invitrogen).

Images were collected using a Zeiss Axiovert 200 M fitted with an AxioCam MRm and were managed using AxioVision 4.4 software (Carl Zeiss microimaging). Image files were exported to Adobe Photoshop CS (Ver.8.0) for preparation of figures.

### RNA FISH

A direct labeled Spectrum Green probe of BAC clone 2272M5 was prepared by Nick Translation according to the manufacturers instructions (Abbot Molecular). The probe (1 microgram) was ethanol precipitated along with 25 micrograms of human Cot-1 DNA before resuspending in 0.1 ml of Hybrisol VII (MP Biomedicals). A direct labeled Spectrum Red probe of XIST exon 1 was prepared as described previously [17]. Monolayer cells were grown directly on slides before fixing and extracting in 4% formaldehyde 0.1% Triton-X100 1× phosphate buffered saline (PBS) for 10 minutes at room temperature. Slides were washed for 2 minutes each in 1× PBS before dehydration through 70% and 100% ethanol for 2 minutes each and then air-drying. Suspension cells resuspended in 1× PBS were seeded onto poly-L lysine coated slides and incubated at room temperature for 20 minutes before fixing and extracting as above. A 1∶1 mix of the BAC and XIST probes was denatured at 72°C for 5 minutes before placing at 37°C for 30–60 minutes to block repetitive elements. The probe was applied onto cells and sealed with a coverslip and rubber cement at 37°C for 16 hours in a humidified chamber. Slides were washed twice at room temperature in 50% formamide, 2× sodium citrate sodium chloride (SSC), followed by 3 minutes at 37°C in 50% formamide 2×SSC and one wash of 3 minutes at 37°C in 2×SSC before addition of ProLong Gold antifade containing DAPI (Invitrogen).

DXZ4 sub-region direct-labeled FISH probes were prepared from sub-fragment clones of DXZ4. Sub-fragment clones were prepared by PCR amplifying unique regions of DXZ4 (PCR Primers S1) and TA cloning into pCR2.1 (Invitrogen). Probes were prepared as described above, except Cot-1 DNA was not used for precipitation and no blocking of repeats were necessary.

Imaging was performed as described as above.

### Plug preparation

Approximately 4×10^7^ cells were resuspended in 1 ml of L-buffer (100 mM EDTA [8.0], 10 mM Tris-HCl [8.0], 20 mM NaCl), before mixing 1∶1 with 1.0% (w/v) molten low-melt agarose (Biorad). The cell mixture was transferred to plug molds (Biorad) with ∼80 ul of the cell suspension per plug (approximately 1.6×10^6^ cells/plug). Plugs were allowed to set at 4°C for 10 minutes before transfer to 10 volumes of L-buffer containing 1% (w/v) sarkosyl and 1 mg/ml Proteinase-K (Roche) and incubating overnight at 50°C. Plugs were rinsed with water before three washes of one hour each with 50 volumes of TE [8.0]. Plugs were incubated at 50°C for 30 minutes in 10 volumes of TE [8.0] supplemented with 80 ug/ml PMSF (Roche). Plugs were rinsed once more with water before three additional hour-long washes in 50 volumes of TE [8.0] at room temperature before storage at 4°C.

### Pulsed field gel electrophoresis

Agarose embedded DNA was digested with the restriction enzymes given in the legend for the appropriate data figures. All enzymes were obtained from NEB. Each plug was first equilibrated in 300 ul of 1× digest buffer at room temperature for 20 minutes, before replacement of buffer with 100 ul of 1× digest buffer containing 200 units of restriction enzyme. Digests were performed overnight at 37°C. Plugs were loaded onto a 1.0% agarose gel prepared using pulsed field certified agarose (Biorad) in 0.5×TBE. The running conditions (voltage per cm, included angle, run time and switching time) were determined by the auto algorithm function of the CHEF Mapper (Biorad). The following conditions were consistent in each case: 0.5× TBE, 14°C, 1.0% agarose. Separation parameters for the Southern blots in the current manuscript were as follows: Figure 1d: 100–400 kb. Figure 4a: 50–200 kb. Figure 4b: 100–200 kb. Figure 4c: 80–200 kb. Markers were loaded in the outer lanes (NEB, MidRange PFG Markers I and II).

### DXZ4 BAC Characterization

BAC clones were identified that matched DXZ4 at both ends by BLAST using a single DXZ4 monomer sequence (Acc. No. S60754). BACs were obtained from Invitrogen and DNA isolated using the Qiagen plasmid Midi kit. BACs were digested with *Hind*III to confirm presence of a common 3 kb fragment and insert size determined by PFGE. BAC clone 2272M5 (Acc. No. AQ745776) was selected for subcloning and sequencing. Cloning vector pBluescript II was digested with *Hind*III before phosphatase treatment using Calf intestinal phosphatase (NEB). BAC 2272M5 was digested with *Hind*III and fragments subcloned into pBluescript II, before blue-white screening using standard techniques [36]. Plasmid DNA was isolated from individual clones using the QIAprep miniprep kit (Qiagen). Clone inserts were sequenced using the oligonucleotides listed in the Supporting Information (PCR Primers S1). All restriction endonucleases were obtained from New England Biolabs.

### Southern blotting & hybridization

At the end of the PFGE run, the gel was rinsed with water before staining with ethidium bromide (1 ug/ml) at room temperature for 30 minutes. The gel was washed twice with water for 15 minutes each and an image captured. The gel was then treated with 0.25 M HCl for 15 minutes before denaturing for 30 minutes (1.5 M NaCl, 0.5 M NaOH). DNA was transferred to Hybond-N+ (GE Healthcare) overnight by standard Southern blotting [36]. The membrane was rinsed with 2×SSC before baking at 120°C for 30 minutes.

A DXZ4 probe was prepared by PCR amplification of regions of DXZ4 using oligonucleotides listed in the Supporting Information (PCR Primers S1). The PCR products were cleaned (Qiagen) before labeling with DIG-11-dUTP by random priming (Roche). The probes were tested for specificity and detection of the anticipated DNA fragment size on a Southern blot of *Eco*RI digested total genomic DNA.

Hybridization was performed overnight at 60°C using Expresshyb (Clontech). Blots were washed the following day at 60°C using two 8-minute washes in 2×SSC, 0.1%SDS followed by one wash of 8 minutes in 0.2×SSC, 0.1%SDS. The probe was detected using anti-DIG-alkaline phosphatase, blocking, wash and detection buffers according to the manufacturers instructions (Roche). Signals were detected by exposure to photographic film (Kodak).

## Supporting Information

Figure S1 **Comparison of DXZ4 hybridization patterns between PvuII and XbaI PFGE.** Southern blots of PFGE separated DNA from 11 independent individuals cut with either XbaI or PvuII and hybridized with a DXZ4-DIG probe. Recognition sequences for either restriction endonuclease are not present in the DXZ4 array and therefore give near identical hybridizing patterns. Sizes in kb are given to the right of each blot. The first PvuII site is 24 kb closer to the array on the distal edge accounting for the smaller sized hybridizing fragments.
(TIF)

Figure S2 **BAC clone insert size determination.** Ethidium bromide stained 1.0% agarose gel showing restriction endonuclease digestion of DXZ4 BAC clone 2272M5 separated by PFGE. Separation performed at 14°C for 26 hours in 0.5× TBE, separating for 20–200 kb on a CHEF Mapper (Biorad). Markers and sizes are indicated, as are the restriction enzymes used that cut in the vector backbone, but not the DXZ4 array. *Not*I cuts twice in pBeloBAC11, excising the BAC insert, accounting for the smaller fragment size.
(TIF)

Figure S3 **Southern blot of PvuII digested DNA from members of CEPH family 1345.** Southern blot of PFGE separated DNA from CEPH family 1345 digested with *Pvu*II and hybridized with a DXZ4-DIG probe. The top portion of the blot has been darkened in Photoshop in order to clearly see the 284 kb extra band (top arrow) also observed with *Xba*I. The middle arrow points to the additional 234 kb band and the lower arrow points to the additional 227 kb band.
(TIF)

Table S1 **DXZ4 variation in genome build HG19.** Summary of SNPs and microsatellite alleles in complete ∼3 kb DXZ4 monomers that define the DXZ4 array in human genome build hg19. Coordinates of SNPs are given relative to the reference sequence of subclone 35. Variants that do not appear in BAC 2272M5 are highlighted in red (only first appearance in the table is highlighted). The largest allele of the (CT) microsatellite is highlighted in green.
(DOCX)

Table S2 **DXZ4 variation in monomer submitted by Giacalone et al.** Summary of SNPs and microsatellite alleles in the single DXZ4 monomer sequence submitted by Giacalone and colleagues [5] Coordinates of SNPs are given relative to the reference sequence of subclone 35. Variants that do not appear in BAC 2272M5 or hg19 are highlighted in blue.
(DOCX)

PCR Primers S1 **DNA sequence of oligonucleotide primers used in current study.**
(DOCX)
