# Supplementary figures and images for: Variation in Array Size, Monomer Composition and Expression of the Macrosatellite DXZ4
Source: PLoS One. 2011 Apr 22;6(4):e18969. doi: 10.1371/journal.pone.0018969 (PMC3081327; doi:10.1371/journal.pone.0018969)

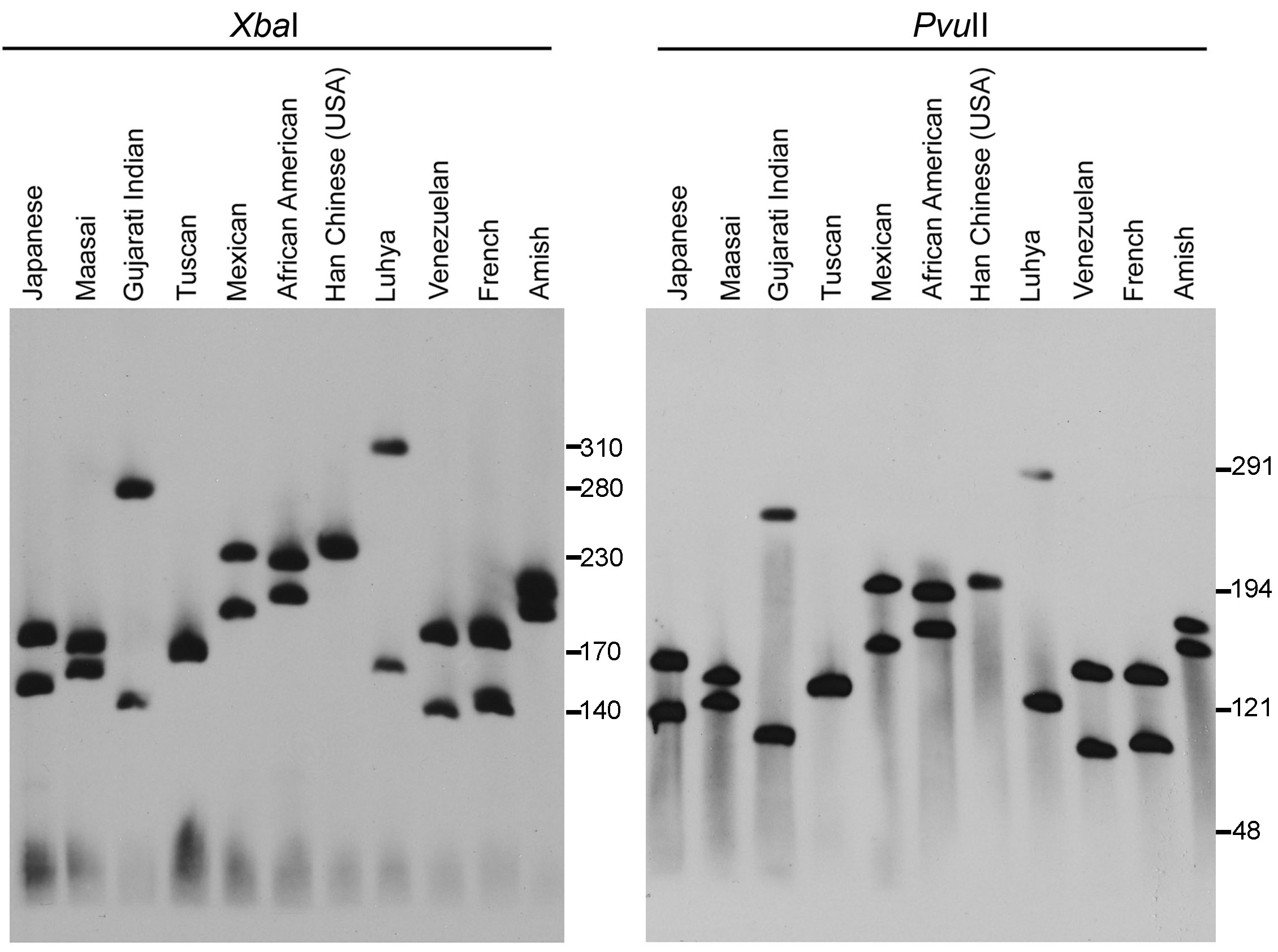

Supplement: Figure S1 — Comparison of DXZ4 hybridization patterns between PvuII and XbaI PFGE. Southern blots of PFGE separated DNA from 11 independent individuals cut with either XbaI or PvuII and hybridized with a DXZ4-DIG probe. Recognition sequences for either restriction endonuclease are not present in the DXZ4 array and therefore give near identical hybridizing patterns. Sizes in kb are given to the right of each blot. The first PvuII site is 24 kb closer to the array on the distal edge accounting for the smaller sized hybridizing fragments. (TIF) [file pone.0018969.s001.tif]

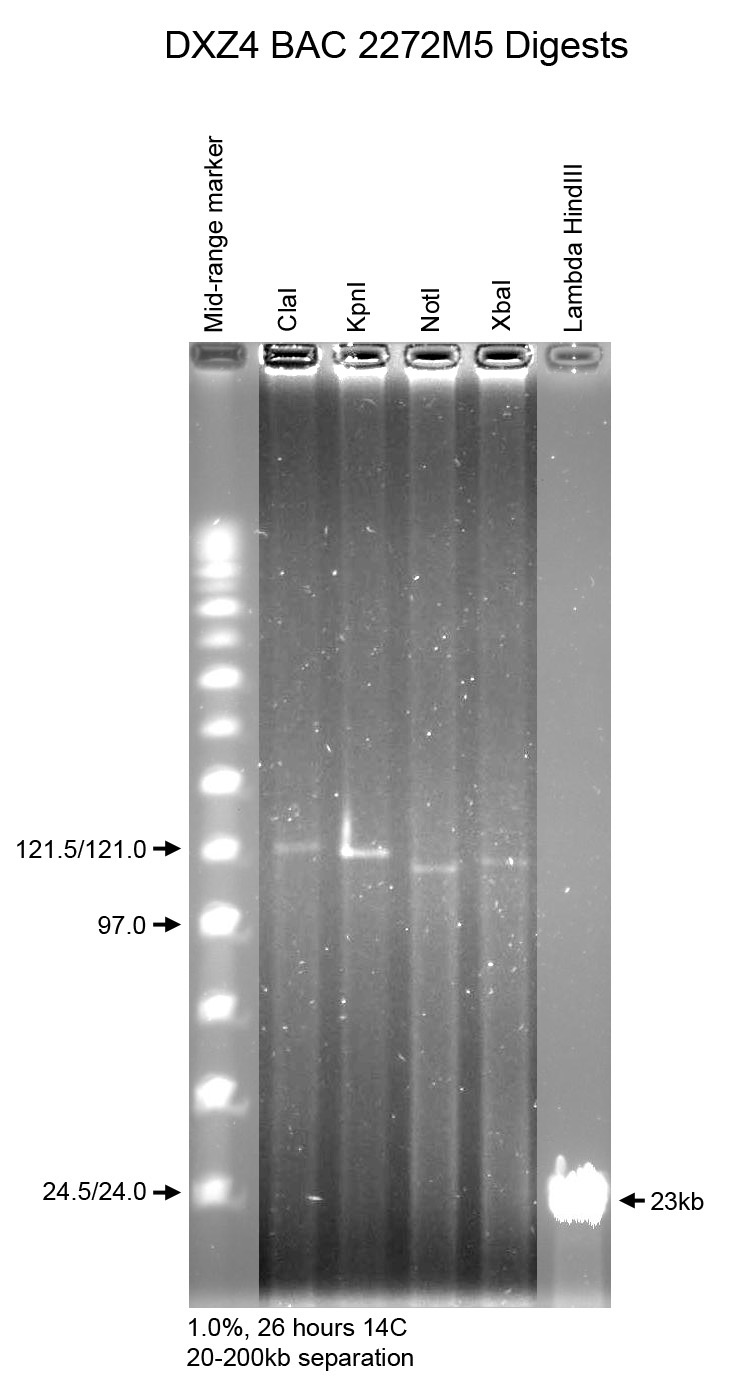

Supplement: Figure S2 — BAC clone insert size determination. Ethidium bromide stained 1.0% agarose gel showing restriction endonuclease digestion of DXZ4 BAC clone 2272M5 separated by PFGE. Separation performed at 14°C for 26 hours in 0.5× TBE, separating for 20–200 kb on a CHEF Mapper (Biorad). Markers and sizes are indicated, as are the restriction enzymes used that cut in the vector backbone, but not the DXZ4 array. NotI cuts twice in pBeloBAC11, excising the BAC insert, accounting for the smaller fragment size. (TIF) [file pone.0018969.s002.tif]

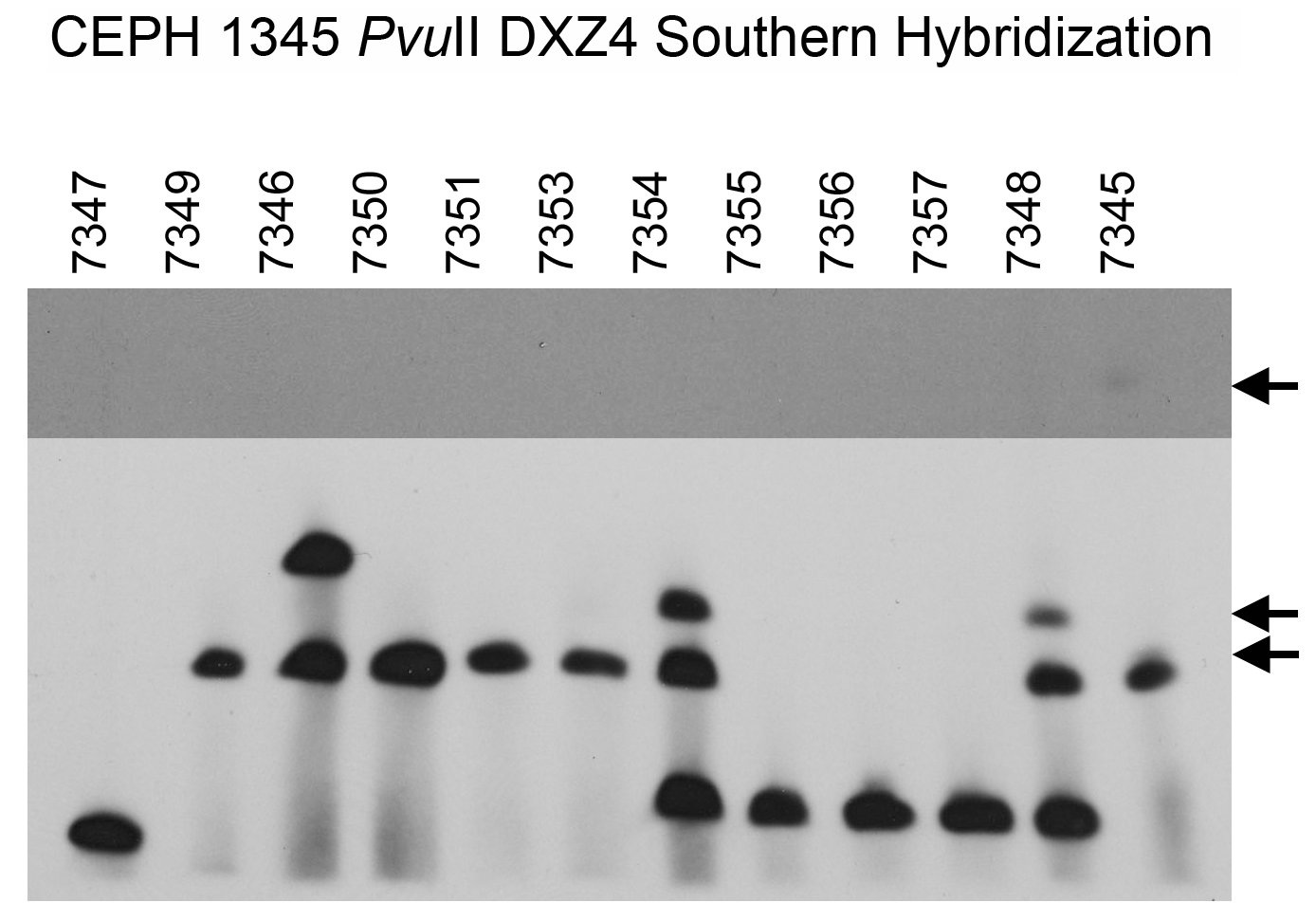

Supplement: Figure S3 — Southern blot of PvuII digested DNA from members of CEPH family 1345. Southern blot of PFGE separated DNA from CEPH family 1345 digested with PvuII and hybridized with a DXZ4-DIG probe. The top portion of the blot has been darkened in Photoshop in order to clearly see the 284 kb extra band (top arrow) also observed with XbaI. The middle arrow points to the additional 234 kb band and the lower arrow points to the additional 227 kb band. (TIF) [file pone.0018969.s003.tif]
