# Supplementary material for: Variation in Array Size, Monomer Composition and Expression of the Macrosatellite DXZ4
Source: PLoS One. 2011 Apr 22;6(4):e18969. doi: 10.1371/journal.pone.0018969 (PMC3081327; doi:10.1371/journal.pone.0018969)
Supplement: Table S1 — DXZ4 variation in genome build HG19. Summary of SNPs and microsatellite alleles in complete ∼3 kb DXZ4 monomers that define the DXZ4 array in human genome build hg19. Coordinates of SNPs are given relative to the reference sequence of subclone 35. Variants that do not appear in BAC 2272M5 are highlighted in red (only first appearance in the table is highlighted). The largest allele of the (CT) microsatellite is highlighted in green. (DOCX) [file pone.0018969.s004.docx]

| **Monomer** | **SNPs** | **(GGGCC)** | **(CT)** | **(TAAA)** |
| --- | --- | --- | --- | --- |
| 1* | ΔG^205^, Ins(GTGGCC)^209^, C-G^364^, A-G^411^, G-A^593^, T-G^1163^, G-C^1890^, A-C^2408^, A-G^2608^, C-T^2656^ | 5 | 11 | 11 |
| 3 | ΔG^205^, Ins(GTGGCC)^209^, C-G^364^, A-G^411^, T-G^569^, G-A^593^, T-G^1163^, C-T^2181^, G-A^2236^, A-G^2608^, C-T^2656^, T-C^2673^, C-G^2813^, G-A^2867^ | 5 | 12 | 11 |
| 4 | Ins(GTGGCC)^209^, C-G^364^, A-G^411^, G-A^593^, T-G^1163^, A-G^2608^, C-T^2656^ | 5 | 14 | 9 |
| 5 | Ins(GTGGCC)^209^, C-G^364^, A-G^411^, G-A^593^, T-G^1163^, T-A^1560^, G-C^1890^, T-G^2485^, A-G^2608^, C-T^2656^, C-G^2813^, G-A^2956^ | 5 | 16 | 11 |
| 6 | Ins(GTGGCC)^209^, C-G^364^, A-G^411^, G-A^593^, T-G^1163^, G-C^1890^, A-C^2408^, A-G^2608^, C-T^2656^ | 5 | 16 | 11 |
| 7 | Ins(GTGGCC)^209^, C-G^364^, A-G^411^, G-A^593^, T-G^1163^, C-T^2181^, A-G^2608^, C-T^2656^ | 5 | 14 | 10 |
| 8 | Ins(GTGGCC)^209^, C-G^364^, A-G^411^, G-A^593^, T-G^1163^, G-C^1890^, A-G^2608^, C-T^2656^ | 5 | 19 | 8 |
| 9 | Ins(GTGGCC)^209^, C-G^364^, A-G^411^, G-A^593^, T-G^1163^, G-C^1890^, A-C^2408^, A-G^2608^, C-T^2656^ | 5 | 14 | 9 |
| 10 | Ins(GTGGCC)^209^, C-G^364^, A-G^411^, G-A^593^, T-G^1163^, T-A^1560^, G-C^1890^, A-C^2408^, A-G^2608^, C-T^2656^ | 5 | 16 | 10 |
| 11 | Ins(GTGGCC)^209^, C-G^364^, A-G^411^, G-A^593^, T-G^1163^, G-A^1826^, G-C^1890^, A-G^2608^, C-T^2656^ | 5 | 11 | 8 |
| 12 | Ins(GTGGCC)^209^, C-G^364^, A-G^411^, G-A^593^, T-G^1163^, G-C^1890^, Ins(CTTTCTCTTTCTCT)^1844^, G-C^1890^, A-G^2608^, C-T^2656^ | 5 | 16 | 8 |
| 13 | Ins(GTGGCC)^209^, C-G^364^, A-G^411^, G-A^593^, T-G^1163^, G-A^1178^, Ins(CTTTCTCTTTCTCT)^1844^, G-C^1890^, A-G^2608^, C-T^2656^ | 5 | 16 | 12 |

*Monomer 1 and 2 are 100% identical (reading from proximal to distal)
