# Supplementary material for: Variation in Array Size, Monomer Composition and Expression of the Macrosatellite DXZ4
Source: PLoS One. 2011 Apr 22;6(4):e18969. doi: 10.1371/journal.pone.0018969 (PMC3081327; doi:10.1371/journal.pone.0018969)
Supplement: Table S2 — DXZ4 variation in monomer submitted by Giacalone et al. Summary of SNPs and microsatellite alleles in the single DXZ4 monomer sequence submitted by Giacalone and colleagues [5] Coordinates of SNPs are given relative to the reference sequence of subclone 35. Variants that do not appear in BAC 2272M5 or hg19 are highlighted in blue. (DOCX) [file pone.0018969.s005.docx]

| **Monomer** | **SNPs** | **(GGGCC)** | **(CT)** | **(TAAA)** |
| --- | --- | --- | --- | --- |
| Giacalone | G-C^51^, A-G^207^, G-C^247^, C-G^364^, C-T^443^, Ins(CCCC)^446^, Ins(CCG)^450^, G-C^480^, C-A^481^, G-A^593^, Ins(C)^724^, ΔC^1669^, G-C^1882^, G-C^1890^, C-T^2307^, Ins(C)^2330^, C-T^2656^, G-A^2736^, C-G^2777^ | 5 | 18 | 8 |
