# Supplementary material for: Variation in Array Size, Monomer Composition and Expression of the Macrosatellite DXZ4
Source: PLoS One. 2011 Apr 22;6(4):e18969. doi: 10.1371/journal.pone.0018969 (PMC3081327; doi:10.1371/journal.pone.0018969)
Supplement: PCR Primers S1 — DNA sequence of oligonucleotide primers used in current study. (DOCX) [file pone.0018969.s006.docx]

***DNA sequence of oligonucleotide primers used in current study***

***Q.RT-PCR Primers***

The following two sets of primers were used to assess DXZ4 expression by quantitative RT-PCR.

Fwd.4 – Rev.19 90bp

Fwd.4 TTAAAGGTGCGTTGACGTGG

Rev.19 GCCAGGGGGATAGGTGTG

Fwd.11 – Rev.22 65bp

Fwd.11 CTACGCCAAGCTGCTAACTC

Rev.22 CCAGCGGAAAGTCCATGGG

***RT-PCR Primers***

The following primer set was used to assess DXZ4 expression by RT-PCR in a panel of human tissue cDNA.

Fwd.19 – Rev.19 253bp

Fwd.19 GAGATGCCCATGAACTCAAG

Rev.19 GCCAGGGGGATAGGTGTG

***PFGE Southern Hybridization***

The following primer sets were used to generate PCR products that were pooled and used to generate a DIG-labeled probe that was hybridized to PFGE Southern blots.

Fwd.6 – Rev.3 485bp

Fwd.6 CAGGCAGAAATGAGCACCAC

Rev.3 TGGTGGCGGCCATGATCTG

Fwd.3 – Rev.1 550bp

Fwd.3 ACCAGGCAAACTGCCCAAG

Rev.1 TTCTGGTTTGTCAGGAAGGC

Fwd.15 – Rev.17 491bp

Fwd.15 ACCCTGTCCTTGGCAGATG

Rev.17 GTTGGACGTAGGCCAGGTG

Fwd.9 – Rev.22 402bp

Fwd.9 GCCTACGTCACGCAGGAAG

Rev.22 CCAGCGGAAAGTCCATGGG

Fwd.5 – Rev.20 488bp

Fwd.5 CACTTGGGAGACTCCTGAAC

Rev.20 TGTCCCCGAGGTTGTCTTG

Fwd.10 – Rev.10 530bp

Fwd.10 TCTCTCGCCCACTTCTACTG

Rev.10 GAGTCGATGGGCCTCTTAG

***Fiber FISH Probes***

The following oligos were used to generate a PCR product that was TA cloned and used to make direct-labeled FISH probes for DNA Fiber FISH.

Fwd.3 – Rev.1 550bp (Spectrum Green Probe)

Fwd.3 ACCAGGCAAACTGCCCAAG

Rev.1 TTCTGGTTTGTCAGGAAGGC

Fwd.10 – Rev.4 449bp (Spectrum Orange Probe)

Fwd.10 TCTCTCGCCCACTTCTACTG

Rev.4 TTCCACCTGAATCCAGCTAG

***RNA FISH Probes***

The following oligos were used to generate a PCR product that was TA cloned and used to make direct-labeled FISH probes for RNA FISH.

Probe-1 Fwd.3 – Rev.1 550bp

Fwd.3 ACCAGGCAAACTGCCCAAG

Rev.1 TTCTGGTTTGTCAGGAAGGC

Probe-2 Fwd.8 – Rev.9 496bp

Fwd.8 TAGGATGGGAGGATGGTGG

Rev.9 ATGTTTGGGCAGGAAGATCG

Probe-3 Fwd.10 – Rev.4 449bp

Fwd.10 TCTCTCGCCCACTTCTACTG

Rev.4 TTCCACCTGAATCCAGCTAG

Probe-4 Fwd.13 – Rev.6 331bp

Fwd.13 CGTCTGCTGCTGTACCAGAC

Rev.6 TTCCTCCTTGCGTCCTAGG

***DNA sequencing oligonucleotides***

The following oligos were used to sequence pBluescript 2272M5 *Hind*III DXZ4 subclones.

T7 TAATACGACTCACTATAGGG

T3 GCAATTAACCCTCACTAAAGG

Fwd.1 TGATGGCAGTATTGCTCCAG

Rev.1 TTCTGGTTTGTCAGGAAGGC

Fwd.2 GGAAAGCCTATGTTGACCTC

Rev.2 GAGTTAGCAGCTTGGCGTAG

Fwd.3 ACCAGGCAAACTGCCCAAG

Rev.3 TGGTGGCGGCCATGATCTG

Fwd.4 TTAAAGGTGCGTTGACGTGG

Rev.4 TTCCACCTGAATCCAGCTAG
